# Multiparameter phenotyping of platelets and characterization of the effects of agonists using machine learning

**DOI:** 10.1016/j.rpth.2024.102523

**Published:** 2024-07-22

**Authors:** Ami Vadgama, James Boot, Nicola Dark, Harriet E. Allan, Charles A. Mein, Paul C. Armstrong, Timothy D. Warner

**Affiliations:** 1 Centre for Immunobiology, Blizard Institute, Faculty of Medicine and Dentistry, Queen Mary University of London, London, United Kingdom; 2 Genome Centre, Blizard Institute, Faculty of Medicine and Dentistry, Queen Mary University of London, London, United Kingdom

**Keywords:** computational biology, flow cytometry, hemostasis, machine learning, thrombosis

## Abstract

**Background:**

Platelet function is driven by the expression of specialized surface markers. The concept of distinct circulating subpopulations of platelets has emerged in recent years, but their exact nature remains debatable.

**Objectives:**

To design a spectral flow cytometry–based phenotyping workflow to provide a more comprehensive characterization, at a global and individual level, of surface markers in resting and activated healthy platelets, and to apply this workflow to investigate how responses differ according to platelet age.

**Methods:**

A 14-marker flow cytometry panel was developed and applied to vehicle- or agonist-stimulated platelet-rich plasma and whole blood samples obtained from healthy volunteers, or to platelets sorted according to SYTO-13 (Thermo Fisher Scientific) staining intensity as an indicator of platelet age. Data were analyzed using both user-led and independent approaches incorporating novel machine learning–based algorithms.

**Results:**

The assay detected differences in marker expression in healthy platelets, at rest and on agonist activation, in both platelet-rich plasma and whole blood samples, that are consistent with the literature. Machine learning identified stimulated populations of platelets with high accuracy (>80%). Similarly, machine learning differentiation between young and old platelet populations achieved 76% accuracy, primarily weighted by forward scatter, cluster of differentiation (CD) 41, side scatter, glycoprotein VI, CD61, and CD42b expression patterns.

**Conclusion:**

Our approach provides a powerful phenotypic assay coupled with robust bioinformatic and machine learning workflows for deep analysis of platelet subpopulations. Cleavable receptors, glycoprotein VI and CD42b, contribute to defining shared and unique subpopulations. This adoptable, low-volume approach will be valuable in deep characterization of platelets in disease.

## Essentials

• Platelet function is directed by the expression of specialized surface markers.
• Circulating platelet subpopulations are incompletely characterized.
• Multiparameter spectral flow cytometry allows robust and comprehensive phenotyping of platelets.
• Coupling with machine learning offers a powerful method to determine platelet subpopulations.

## 1 Introduction

Hemostasis is a carefully orchestrated process in which platelets are primary players. These metabolically active cell fragments circulate for approximately 7 to 10 days in healthy individuals before being degraded by the spleen or liver [[1], [2], [3]]. Platelet function is mediated through the expression of specialized surface markers, with resting and activated platelets showing different expression profiles consistent with response heterogeneity in thrombus formation [4,5]. These variations in surface marker expression are thought to be conferred during production from megakaryocytes, activation history, and aging in the circulation [6,7], and may describe dynamically discrete and specialized platelet subpopulations [8,9].

As platelets age in the circulation, they lose mRNA remaining from their progenitor megakaryocytes [[10], [11], [12]]. Platelets have minimal capacity to make new mRNA, and thus, these residual mRNAs can be used as a surrogate measure for age [[13], [14], [15]]. Newly formed or “young” platelets (also termed reticulated platelets or the immature platelet fraction) have the highest levels of mRNA, while “old” platelets have the lowest [14,16,17]. Young platelets are hyperreactive and have an elevated thrombotic potential [[18], [19], [20]]. This is apparent in several pathologic states, such as diabetes mellitus [21] and major trauma [18], in which there are relative increases in young platelets resulting from altered platelet turnover and lifespan associated with increased incidences of thromboembolic events [[22], [23], [24], [25]] and decreased efficacy of antiplatelet therapies [[26], [27], [28], [29]].

Flow cytometry with the inclusion of fluorescently tagged antibodies is frequently used to investigate platelet protein expression and function as it requires only small volumes of blood and a relatively low number of platelets, making it ideal for analysis of clinical samples. However, the number of parameters that can be measured concurrently using this method is limited by overlap of fluorescent emission spectra, resulting in antibody panels that typically determine, at most, 3 to 4 markers in any 1 sample [30,31]. Spectral flow cytometry is a next-generation technique that allows the simultaneous measurement and discrimination of multiple fluorophores by evaluation of full emission spectral signatures. Accounting for steric hindrance, this platform can therefore be used for analysis of 10 to 15 markers on platelets. Previous work from Blair et al. [8] has used mass cytometry to develop a panel to study platelet function, but spectral flow cytometry offers several considerable advantages including cost of reagents, availability of equipment, and simplicity of technique.

With this in mind, we sought to develop a 14-marker spectral flow cytometry panel based upon the panel of Blair et al. [8] (noting in this report that Blair et al. [8] have subsequently published reports utilizing spectral flow cytometry [30,32]) to provide a more comprehensive characterization of platelets at a global and individual platelet level. In our studies, we subjected our data to a powerful computational analytical approach employing machine learning (ML) to explore the potential existence of platelet subpopulations in the human circulation with a particular focus on young and old platelets.

## 2 Methods

### 2.1 Ethical statement: human studies

All studies were conducted according to the principles of the Declaration of Helsinki and were approved by St. Thomas’ Hospital Research Ethics Committee (reference number: 07/Q0702/24). Healthy volunteers were aged 23 to 40 years, were screened prior to entering the study (were nonsmokers, had not taken nonsteroidal anti-inflammatory drugs ≤10 days prior to donating blood, and had no health problems contraindicating study involvement), and gave written informed consent.

### 2.2 Collection of blood and preparation of platelet-rich plasma

Blood was drawn from volunteers by venipuncture into trisodium citrate vacutainers (3.2%; BD Biosciences), and platelet-rich plasma (PRP) was isolated as previously published [24].

### 2.3 Flow cytometric measurement of activation markers

PRP, or whole blood (WB), was diluted 1:40 with 2 mM Ca^2+^-buffered, filtered phosphate-buffered saline and added to a 96-well plate with wells containing vehicle (phosphate-buffered saline) or agonist: 0.3 to 30 μM adenosine diphosphate (ADP; Labmedics); 0.3 to 30 μM thrombin-receptor activating peptide 6 (TRAP-6; Cambridge Biosciences); 0.3 to 30 μM U46619 (Enzo Life Sciences); 3 to 100 μM protease-activated receptor (PAR) 4 agonist (Cambridge Biosciences); and 0.03 to 3 μM cross-linked collagen-related peptide (CRP-XL; University of Cambridge). In initial experiments, an antibody mix comprising anti-CD42b-BV421 (1:70; clone HIP1; BioLegend), PAC-1-FITC (1:10; BD Biosciences), and anti-CD62P-APC (1:100; clone AK4; BioLegend) was added to each well. Alternatively, an antibody master mix (Supplementary Table S1 and Supplementary Figure S1) and staining buffer (Brilliant Stain Buffer, BD Biosciences) were used. The plate was then mixed (200 rpm; 37 °C) for 20 minutes in the dark (BioShake iQ, Quantifoil Instruments GmbH). Samples were fixed with 1% formalin and run on the Cytek Aurora 5 laser flow cytometer (Cytek Biosciences). Platelets were gated on side scatter area (SSC-A)/CD42b-A, and 10,000 CD42b+ events were collected (Supplementary Figure S2).

### 2.4 Flow cytometric sorting of young and old platelets

Adapting our previously published approach [33], PRP was stained with SYTO-13 (750 nM; Thermo Fisher Scientific) prior to sorting. Ten million platelets per condition were sorted using a BD FACSAria III Fusion Cell Sorter (70 μM nozzle, 70 ps, ≤10,000 events/s; BD Biosciences) with the top 20% SYTO-13 fluorescence being taken as “young” and the bottom 30% SYTO-13 as “old”. Platelets were pelleted in the presence of prostacyclin (epoprostenol [2μmol/L]; Tocris Biosciences) at 1000 × *g* for 10 minutes and resuspended in calcium chloride (CaCl_2_; 2 mmol/L; Sigma-Aldrich)–containing HEPES-buffered modified Tyrode's buffer. Panel markers were then measured as described above using the Cytek Aurora.

### 2.5 Statistical, bioinformatics, and ML analyses

Data were collected using SpectroFlo v2 (Cytek Biosciences) software and analyzed using NovoExpress 1.3.0 (ACEA Biosciences Inc), FlowJo v10 (TreeStar Inc), and GraphPad Prism 9 (GraphPad Software Inc) software. Data were expressed as median fluorescence intensity (MFI) ± SEM and analyzed with a 1-/2-way analysis of variance or mixed-effects analysis followed by multiple-comparison post-hoc tests, as appropriate. Correlations were assessed by nonlinear regression. Statistical significance was assumed for *P* < .05.

Bioinformatic analysis was performed using R version 4.2 or later. Data were loaded into an RStudio (2022.02.2) environment using a loading function from the flowCore (v2.10.0 or later) package [34,35]. Data loading checks for quality control purposes were performed by checking the correlations between MFIs of all parameters in the loaded data and FlowJo v10-analyzed data. Data were further analyzed using the Spectre package [36] and associated functions. Namely, the data were transformed using logicle transformation, and then dimensionality reduction was performed using principal component analysis followed by *t*-distributed stochastic neighbor embedding (tSNE; an unsupervised, nonlinear dimensionality reduction technique for visualizing high-dimensional data in a 2- or 3-dimensional space). Individual platelets are represented as single points and grouped together based on their degree of similarity of expression patterns of all 14 to 16 parameters. tSNE also identifies heterogeneity in platelet responses, allowing the identification of subpopulations of platelets. FlowSOM, a self-organizing map clustering algorithm, was used for cluster generation, which automatically determines the optimal number of clusters for the dataset.

Using the Caret v6.0-93 (Classification And REgression Training) R package [37], we developed a ML model to predict whether platelets were treated with the vehicle or agonist. Platelet data were loaded and processed as already detailed, with 10,000 platelets per healthy donor per treatment being loaded, unless otherwise stated. Balanced in number for each condition, from 16 donors, a random forest model was given a training dataset made up of 80% of the total data to learn from. The remaining 20% of the total data were used for preliminary validation. The remaining 5 donors were used as an “unseen” validation dataset to test the model. A feature importance comparison was then run to determine which markers were most important in the classification. The model was trained using a 10-fold cross-validation using 3 repeats. This means that the training data are first randomly shuffled and split into 10 “folds”; then, in turn, each fold is excluded from the training data while the remaining 9 folds are used to train a model; the excluded fold is used to assess the accuracy of the trained model. This process was then repeated a total of 3 times; each time, the data were shuffled randomly, producing new folds. The metric used to assess the quality of the model was the receiver operating characteristic; overall accuracy was also reported.

Using the Caret v6.0-93 (Classification And REgression Training) R package [37], we developed random forest ML models to predict whether platelets were young or old in the presence of vehicle or individual agonists. Platelet data were loaded and processed as already detailed, with 9000 platelets per young and per old sample from each donor being loaded, outlier events/platelets were removed, and the measurements for PAC-1 excluded. In total, there were 8 donors, each with a young and old sample. Six of the 8 donors were used to create a training and preliminary validation dataset; 80% of the data from the 6 donors were used for training while 20% were used for preliminary validation to assess the accuracy of the model. Data from the remaining 2 donors were kept aside to use as an “unseen” validation dataset. As previously, a feature importance comparison was then run to determine which markers were most important in the classification. The model was trained using a 10-fold cross-validation using 3 repeats, and the receiver operating characteristic was used to assess the quality of the model, as already detailed.

## 3 Results

### 3.1 Assay capable of detecting significant changes in marker expression upon activation in healthy platelets

To select optimum concentrations of agonists for use in subsequent experiments, flow cytometry was used to assess changes in PAC-1 binding and cluster of differentiation (CD)62P expression in response to increasing concentrations of TRAP-6, PAR-4, CRP-XL, ADP, and U46619 (Supplementary Figure S3). The following concentrations were selected for their ability to induce a robust response: 10 μM TRAP-6, 100 μM PAR-4, 3 μM CRP-XL, 30 μM ADP, and 10 μM U46619.

**Figure 1:**
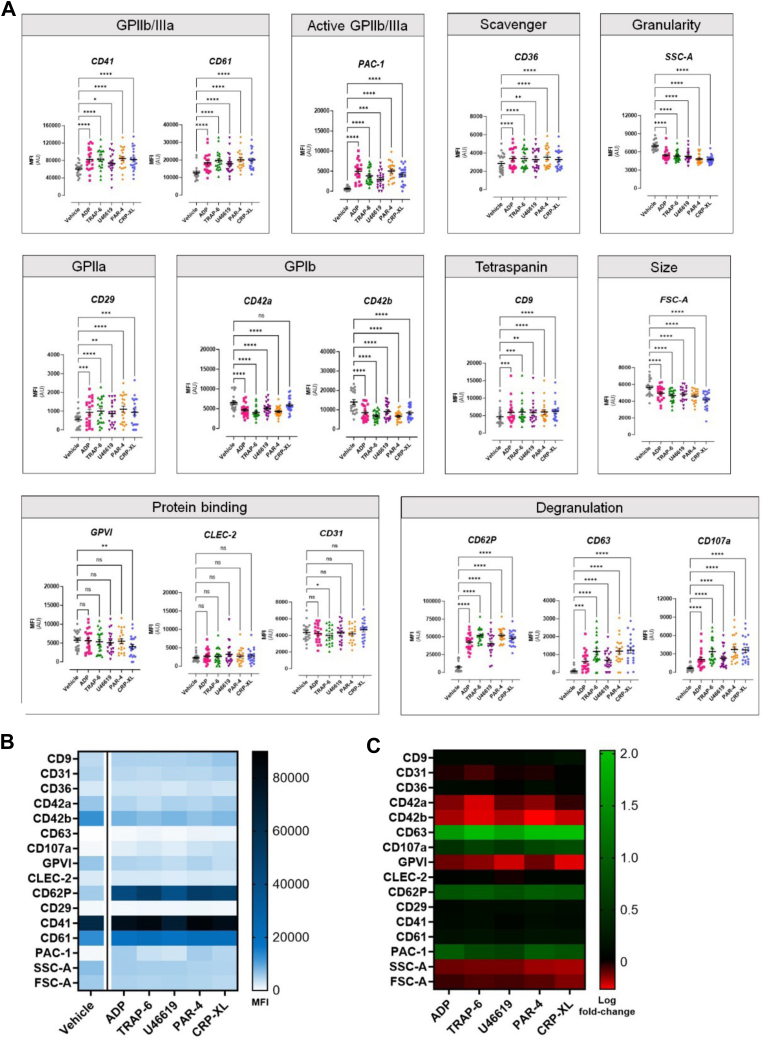
Changes in individual surface marker expression in resting and activated platelets. Platelets were incubated with vehicle (phosphate-buffered saline), adenosine diphosphate (ADP; 30 μM), thrombin-receptor activating peptide 6 (TRAP-6; 10 μM), U46619 (10 μM), protease-activated receptor 4 agonist (PAR-4; 100 μM), or collagen-related peptide (CRP-XL; 3 μM) for 20 minutes at 37 °C. Results are expressed as follows: (A) raw median fluorescence intensity (MFI) ± SEM were analyzed using a 1-way analysis of variance with a Dunnett test to correct for multiple comparisons. ∗*P* < .05, ∗∗*P* < .01, ∗∗∗*P* < .001, ∗∗∗∗*P* < .0001 or non-significant (ns). (B) A heatmap based on MFI values. (C) A heatmap based on MFI log fold changes. All data were obtained from 20 to 21 volunteers. GP, glycoprotein; FSC-A forward scatter; SSC-A, side scatter; CD, cluster of differentiation; CLEC-2, C-type lectin-like receptor 2.

The chosen concentrations of agonists were then tested using the extended phenotyping panel of markers as well as forward scatter area (FSC-A) and SSC-A. All agonists tested caused significant increases in the expression of CD62P, PAC-1, CD63, CD107a, CD61, CD29, and CD9 and decreases in CD42b; none of the agonists caused any changes in C-type lectin-type receptor (CLEC)-2 (Figure 1). TRAP-6 was the only agonist to decrease CD31 (4336 ± 233 vs 3973 ± 223; *P* = .03), and CRP-XL was the only agonist to decrease glycoprotein (GP) VI (5735 ± 512 vs 4030 ± 600; *P* = .006). CD42a expression was decreased by all agonists except CRP-XL. Across all conditions, the physical characteristics FSC-A and SSC-A demonstrated the least interindividual variation (Supplementary Figure S4).

### 3.2 High-dimensionality analysis allows visualization and in-depth interrogation of marker changes in response to activation

**Figure 2:**
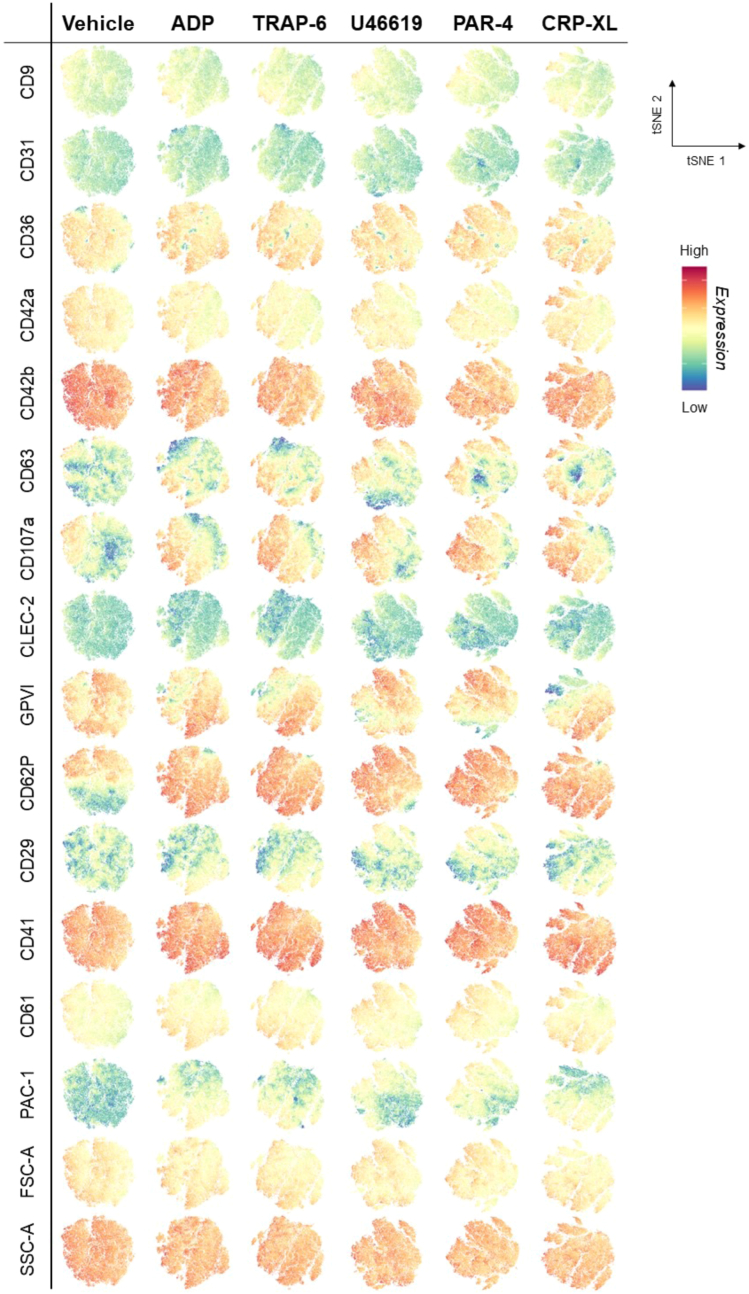
*t*-distributed stochastic neighbor embedding (tSNE) visualization of concatenated samples. Plots calculated for combined datasets (n = 20-21) of platelets incubated with (columns) vehicle (phosphate-buffered saline), adenosine diphosphate (ADP; 30 μM), thrombin-receptor activating peptide 6 (TRAP-6; 10 μM), U46619 (10 μM), protease-activated receptor 4 agonist (PAR-4; 100 μM), or collagen-related peptide (CRP-XL; 3 μM) for 20 minutes at 37 °C**.** Each plot is individually colored to reflect intensity (low = blue; high = red) for each of the measured 16 parameters. GP, glycoprotein; FSC-A forward scatter; SSC-A, side scatter; CD, cluster of differentiation; CLEC-2, C-type lectin-like receptor 2.

Unsupervised dimensionality reduction and visualization of the entire datasets using *t*-distributed stochastic neighbor embedding (tSNE) revealed the same shifts in receptor patterns. Namely, agonist stimulation caused visible increases in the expressions of PAC-1, CD62P, CD63, CD107a, CD61, and CD9 and decreases in the expressions of in CD42b, CD42a, GPVI, CLEC-2, and CD31; CD29 expression remained unchanged (Figure 2). The same visualization approach also confirmed that the detected marker expression patterns were shared across donors and not driven by donor or batch effect (Supplementary Figure S5). Autoclustering analysis produced 5 clusters in vehicle-treated platelets and between 9 and 11 clusters in agonist-treated platelets. Each formed cluster was not dominated by individual donors but rather reflected the gradation in difference of expression (Supplementary Figure S6). However, hierarchical dendrograms within the clustering indicated that interlinked relationships between the expression of each marker differed by agonist stimulation (Supplementary Figure S6).

### 3.3 ML reveals the most important markers in distinguishing effects of agonists

ML was used to further analyze the data at the single platelet level in an unbiased fashion. Following training, the accuracy of differentiation of vehicle-treated from agonist-treated platelets was determined. Accuracy rates for unseen datasets were highest for TRAP-6, PAR-4, and CRP-XL at 0.92, 0.91, and 0.88, respectively. Comparatively, rates for ADP and U46619 were 0.80 and 0.77, respectively (Table 1), with the greatest fall between the training and unseen sets also occurring with these agonists.

**Table 1:** Predictive efficacy of machine learning for stimulated platelets.

Test set	ADP	TRAP-6	U46619	PAR-4	CRP-XL
Training set accuracy	0.92	0.94	0.89	0.95	0.95
Unseen set accuracy	0.80	0.90	0.78	0.87	0.89

ADP, adenosine diphosphate; CRP-XL, collagen-related peptide; PAR-4, protease-activated receptor 4 agonist; TRAP-6, thrombin-receptor activating peptide 6.

**Figure 3:**
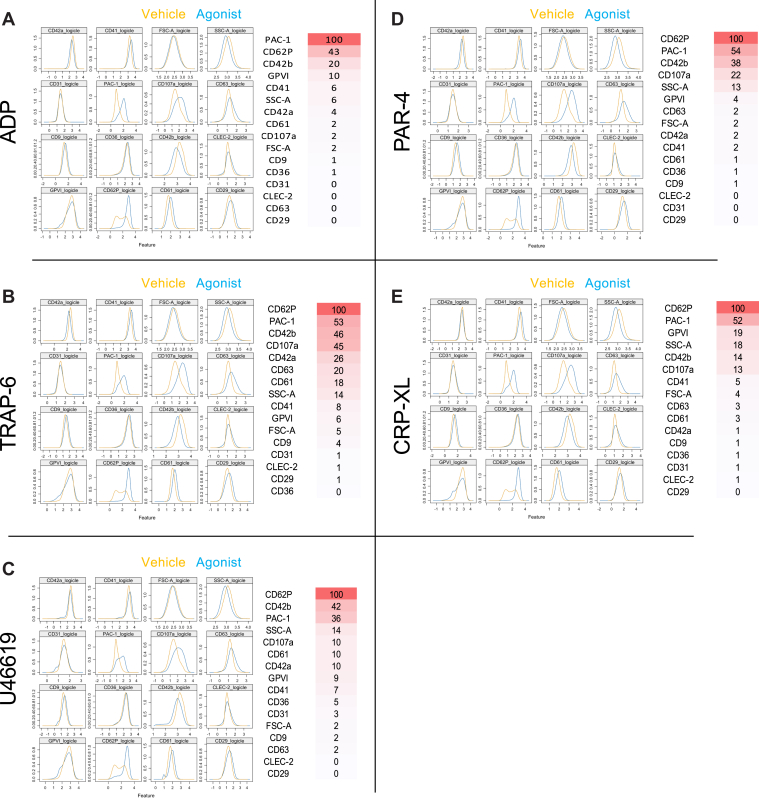
Marker weighting from machine learning predications to distinguish between platelets incubated with vehicle and agonists. (A) Adenosine diphosphate (ADP; 30 μM), (B) thrombin-receptor activating peptide 6 (TRAP-6; 10 μM), (C) U46619 (10 μM)**,** (D) protease-activated receptor 4 agonist (PAR-4; 100 μM), and (E) collagen-related peptide **(**CRP-XL; 3 μM**)**. (Left) Visual comparison of logicle transformed expression data and (right) marker weighting listed in order of importance with gradient overlay representing most important (red) and least important (white; ***n* = 20-21 for all)**. GP, glycoprotein; FSC-A forward scatter; SSC-A, side scatter; CD, cluster of differentiation; CLEC-2, C-type lectin-like receptor 2.

Rankings of distinguishing markers within each prediction model were examined with those with a weighting of greater than 20 considered important (Figure 3). For identification of TRAP-6–, PAR-4–, and CRP-XL–stimulated platelets, CD62P was considered the most important, followed by PAC-1, CD42b, and CD107a (Figure 3A). Identification of ADP-treated platelets was based predominantly on the expression patterns of PAC-1, CD62P, and CD42b (Figure 3B–D). This was similar to platelets activated with U46619, for which CD62P, CD42b, and PAC-1 were highest (Figure 3E).

### 3.4 CD41/CD61, FSC-A, SSC-A, GPVI, CLEC-2, and CD61 are the primary markers used by ML to differentiate between young and old platelets

Next, we undertook phenotypic analysis of young and old platelets, as determined by SYTO-13 RNA staining, following vehicle or agonist stimulation. We first analyzed the data on a traditional by-population basis by looking at raw median fluorescence intensity values and changes in median fluorescence intensity (Supplementary Figure S7) before applying ML. Predictive capability within each training set ranged from 0.86 to 0.90 and maintained high level of accuracy in the unseen datasets (range, 0.74-0.78; Table 2).

**Table 2:** Predictive efficacy of machine learning for young and older platelets.

	Vehicle	TRAP-6	PAR-4	CRP-XL	ADP	U46619
Training set accuracy	0.86	0.87	0.86	0.86	0.90	0.86
Unseen set accuracy	0.76	0.78	0.77	0.76	0.76	0.74

ADP, adenosine diphosphate; CRP-XL, collagen-related peptide; PAR-4, protease-activated receptor 4 agonist; TRAP-6, thrombin-receptor activating peptide 6.

**Figure 4:**
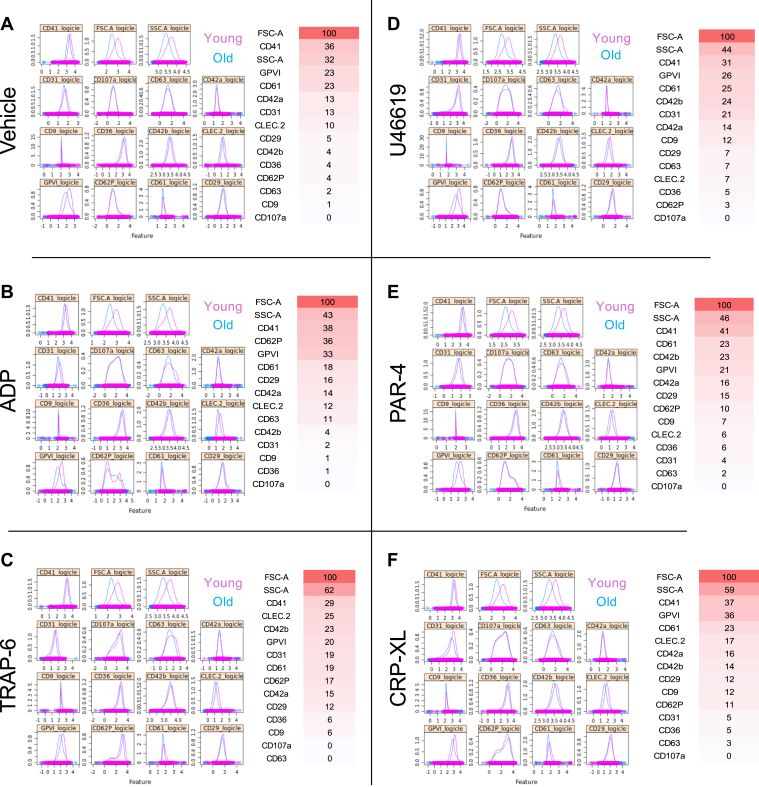
Marker weighting from machine learning predications to differentiate between “young” and “old” platelets incubated (20 minutes at 37 °C) with (A) vehicle (phosphate-buffered saline), (B) adenosine diphosphate (ADP; 30 μM), (C) thrombin-receptor activating peptide 6 (TRAP-6; 10 μM), (D) U46619 (10 μM), (E) protease-activated receptor 4 agonist (PAR-4; 100 μM), and (F) collagen-related peptide (CRP-XL; 3 μM). (Left) Visual comparison of logicle transformed expression data and (right) marker weighting listed in the order of importance with gradient overlay representing most important (red) and least important (white; *n* = 8 for all). GP, glycoprotein; FSC-A forward scatter; SSC-A, side scatter; CD, cluster of differentiation; CLEC-2, C-type lectin-like receptor 2.

Markers rated greater than an importance of 20 within vehicle-treated platelets identified FSC-A, CD41, SSC-A, GPVI, and CD61 as most important (Figure 4). FSC-A, SSC-A, and CD41 were the top 3 discriminators for all agonists tested, with CD61, CD42b, and GPVI being also seen at above 20 in all. CLEC-2 was seen in TRAP-6, and CD61 in CRP-XL.

**Figure 5:**
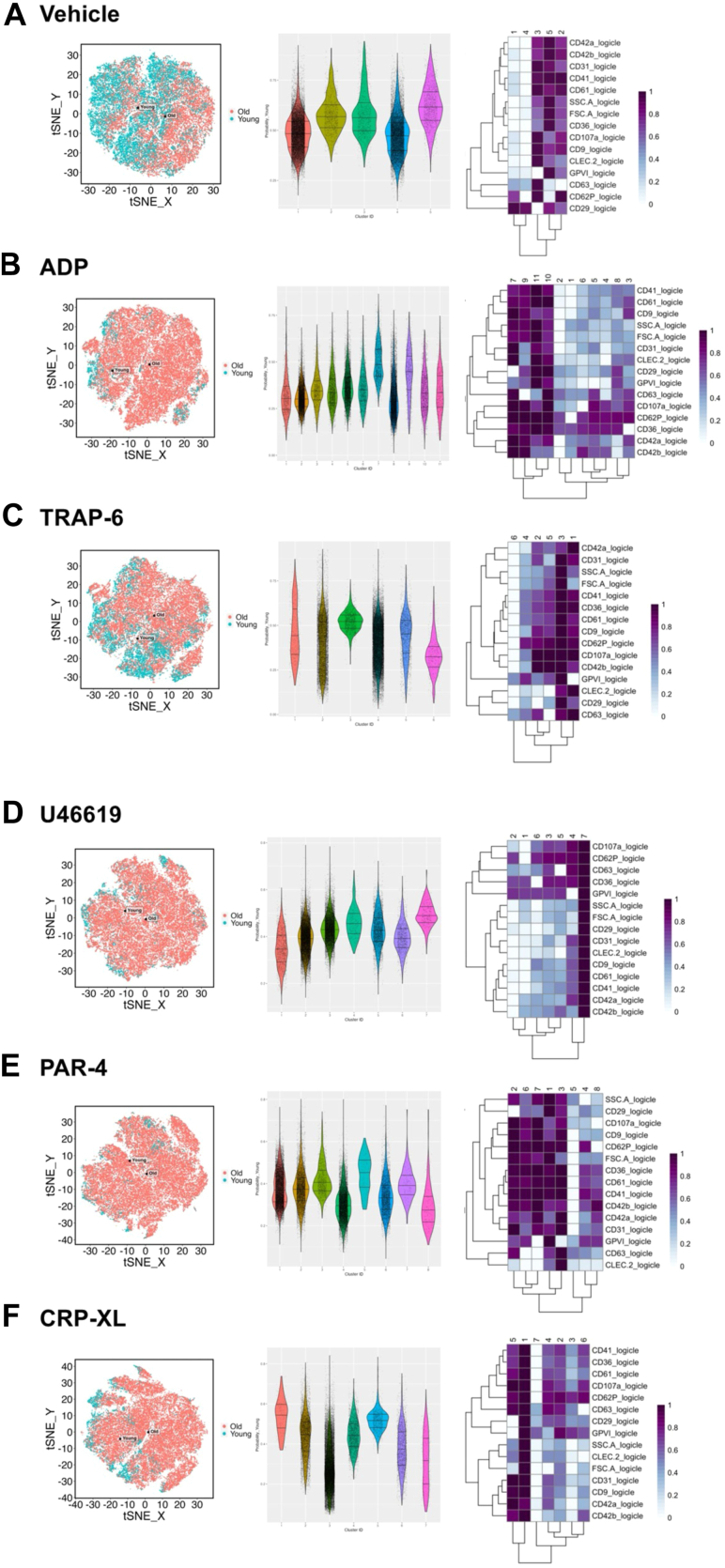
Interrogation of machine learning predicted “young” and “old” platelets within platelet-rich plasma samples incubated with vehicle or agonists. Platelets incubated (20 minutes at 37 °C**)** with (A) vehicle (phosphate-buffered saline), (B) adenosine diphosphate (ADP; 30 μM), (C) thrombin-receptor activating peptide 6 (TRAP-6; 10 μM), (D) U46619 (10 μM), (E) protease-activated receptor 4 agonist (PAR-4; 100 μM), and (F) collagen-related peptide (CRP-XL; 3 μM). (Left) *t*-Distributed stochastic neighbor embedding **(**tSNE) division of concatenated datasets colored with predicted “young” (teal) or “old” (red). (Middle) Violin plot of probability value for each event divided in clusters per condition, as identified by markers weighted by machine learning at above importance. (Right) Heatmap breakdown of relative marker expression within each cluster (low = white; high = purple; *n* = 20-21 for all). GP, glycoprotein; FSC-A forward scatter; SSC-A, side scatter; CD, cluster of differentiation; CLEC-2, C-type lectin-like receptor 2.

Subsequently, we applied the markers rated greater than an importance of 20 from our separated young and old platelet populations to the mixed healthy platelet data presented previously. This analysis indicated an increased probability of young platelets being present in clusters with higher markers of activation, both in control conditions (ie, vehicle-treated) and following exposure to platelet agonists (Figure 5).

### 3.5 Assay detects comparable alterations in marker expression following stimulation and labeling of WB

We next wished to test the suitability of this assay for use with WB, rather than PRP. Agonist stimulation elicited significant increases in the expression of CD62P, PAC-1, CD63, CD107a, CD61, CD29, and CD9 and decreases in CD31 andCD42b; none of the agonists caused any changes in CLEC-2 (Supplementary Figure S8). This pattern of altered marker expression was consistent with those observed in PRP samples, but with a significantly greater loss of CD31 (Supplementary Figure S9).

### 3.6 High-dimensionality analysis of stimulated WB samples reveals 3 common subpopulations of activated platelets

**Figure 6:**
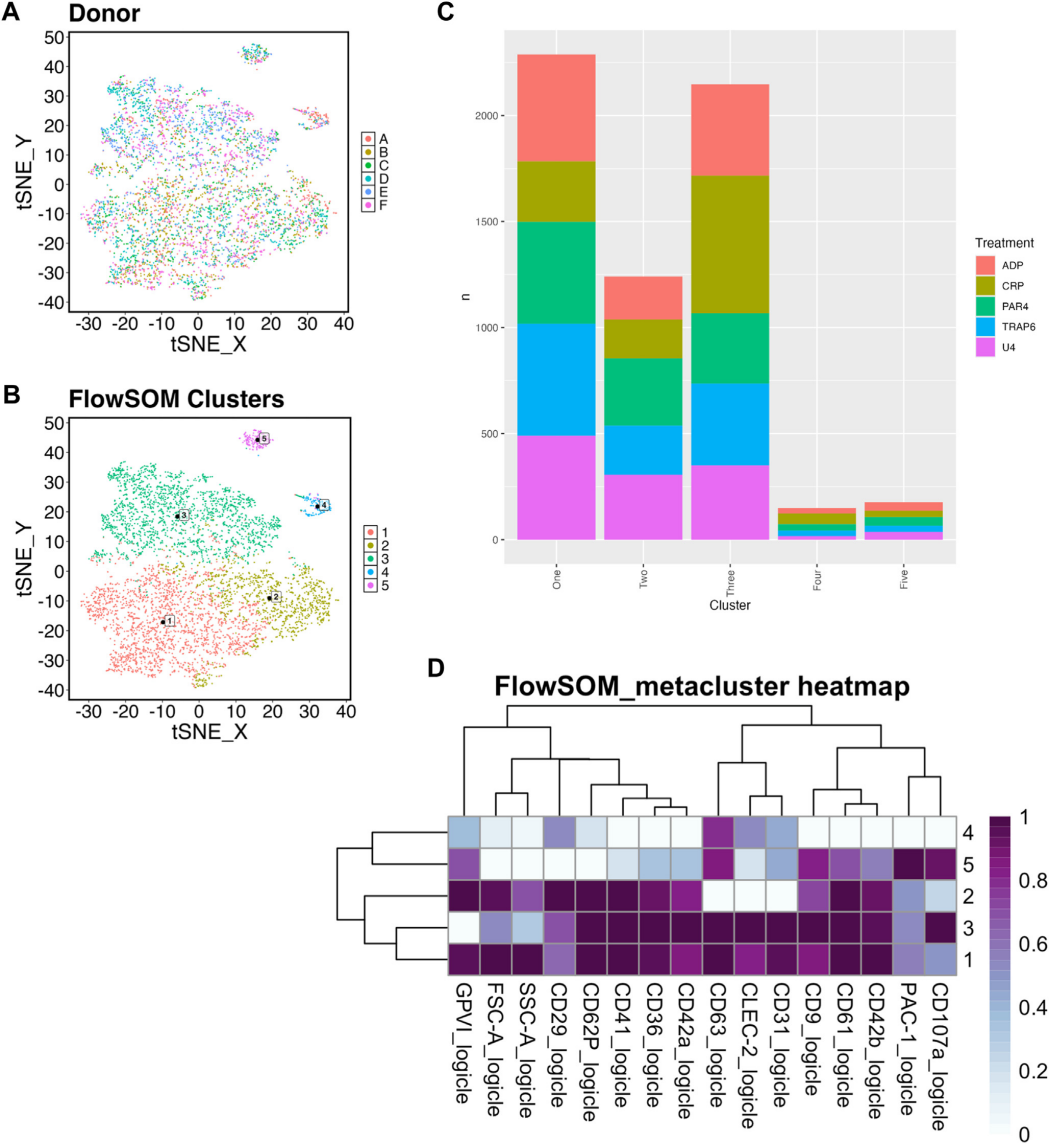
Identification of common subpopulations within whole blood incubated with agonists’ (20 minutes at 37 °C) whole blood. *t***-**Distributed stochastic neighbor embedding **(**tSNE) visualization of concatenated data colored by (A) donor or (B) identified cluster. (C) Relative proportion of clusters and contribution by the agonist (red: adenosine diphosphate **[**ADP]; olive: collagen-related peptide **[**CRP-XL]; green: protease-activated receptor 4 agonist **[**PAR-4]; blue: thrombin-receptor activating peptide 6 **[**TRAP-6]; purple: U46619). (D) Comparative expression heatmaps with hierarchical dendrograms of identified clusters (*n* = 6 for all). GP, glycoprotein; FSC-A forward scatter; SSC-A, side scatter; CD, cluster of differentiation; CLEC-2, C-type lectin-like receptor 2; SOM, self-organizing map.

tSNE-based dimensionality reduction and visualization confirmed that the detected alterations in marker expression (ie, omitting vehicle-treated samples) in WB were again not driven by the donor or batch effect (Figure 6A). Autoclustering analysis of the agonist-stimulated only dataset produced 5 clusters (Figure 6B), with 3 clusters (1, 2, and 3) accounting for more than 90% of total events. Each formed cluster was not dominated by individual agonists, indicating a commonality to these resulting phenotypes. Comparative expression heatmaps with hierarchical dendrograms within the clustering indicated that cluster 2 was characterized by lower expression of CD31, CD63, and CLEC-2, while platelets in cluster 3 had lower FSC-A values, lower levels of GPVI, and higher CD107a expression (Figure 6D).

## 4 Discussion

The levels of individual platelet surface receptors, basally and following activation, have been well-characterized. However, improvements in flow cytometry and mass cytometry technology now permit much larger antibody panels for simultaneous measurement of receptors on individual platelets. Through greater immunophenotyping of platelets in health and in disease, it is increasingly possible to address the question of whether, and what, platelet subpopulations exist. Here we report a 16-parameter phenotypic approach utilizing spectral flow cytometry with computational analysis, including ML, to phenotype platelets.

Our selection of surface proteins was based on the phenotypic panel used by Blair et al. [8] for mass cytometry. First, we set out to measure the effect of different activation pathways of platelet activation on the expression of surface markers. A spectral cytometry–based approach, compared with mass cytometry in terms of antibody costs, machine running costs, and processing time, permits an expansion in experimental conditions that can be performed in each run. Moreover, we established that this assay could be applied to either PRP or WB samples. Across the range of conditions tested (agonists and sample type), there were significant increases in PAC-1, CD62P, CD63, CD107a, CD61, and CD29 expressions; decreases in CD42b expression; and no changes in CLEC-2 expression. These patterns of surface protein changes were consistent with the current understanding of the field and echo those presented by Blair et al. [8] and Hindle et al. [38].

These types of initial analyses rely on median fluorescence scores derived from measurements of 10,000 platelets per subject, and comparative variation determined across donors. Dimensionality reduction (tSNE) and clustering provide for greater analytical weight for each individual platelet across all replicates. It also allows for the visualization of the contribution of each individual subject to the interpreted expression patterns [39]. This approach validated that these observed patterns are shared across donors, confirming that these effects were driven by functional responses and not by potential batch variation. While biological sex has recently been associated with some increased platelet reactivity [40], this shared pattern indicates that there were no obvious sex-based differences in our small group of younger, healthy individuals.

Unlike previous reports [38], based on P-selectin expression and PAC-1 binding, *k*-means clustering silhouette analysis within our dataset did not support the presence of 4 (or more) distinct subpopulations. We did observe that across all agonists, on activation, the cluster of platelets that had the largest drop in GPVI or CD42a/b was not always the same cluster of platelets that had the highest increase in classic activation markers (CD62P/PAC-1). In our WB stimulation experiments, the 3 common clusters that emerged across all agonists had comparable levels of CD62P expression and PAC-1 binding but differed due to loss of GPVI or CD31. This implies that susceptibility to activation-induced “shedding” may be a constituent factor to predestined subpopulations. However, further characterization of these potential subpopulations would be required to determine their composition before activation.

We next turned to ML to construct unbiased algorithms to uncover potential classifications at an individual platelet level and to compare predictive capacities between vehicle- and agonist-treated datasets. Consistent with platelet biology and our traditional user-led population analysis, CD62P expression and PAC-1 binding were consistently identified with agonist stimulation, as was loss of CD42b expression. Interestingly, the weighted importance of CD107a, lysosomal-associated membrane protein 1, was only high for PAR-1 activation using TRAP-6. Using the computed weightings, ML correctly identified over 89% of platelets within the training dataset. When applied to an unseen dataset, correct identification was predictably lower but maintained greater than 77% accuracy, rising to 91% to 92% for PAR-stimulated platelets. The difference in predictive capabilities is likely due to U46619 and ADP being comparatively weaker secondary agonists and therefore producing a less uniform pattern of response [[41], [42], [43], [44]].

We next applied the same ML approach to differentiate between young and old platelets within mRNA stain-based, flow-sorted platelet samples, which were subsequently vehicle- or agonist-stimulated. Strikingly, there is remarkable consistency in the highly weighted parameters across the unstimulated and stimulated samples with FSC-A, CD41, SSC-A, GPVI, CD61, and CD42b featuring prominently.

A significant change in CD41 with platelet age is consistent with previous work from our group looking at the proteomics and transcriptomics of young and old platelets [33], and from others looking at their thrombotic potential [19]. Similarly, an association between GPVI levels and young platelets has been recently reported by Veninga et al. [45] in human platelets and by us using a temporal labeling approach in mice [12].

Interestingly, FSC-A, which is considered an approximate indicator of size in flow cytometry, is also highly important in our ML algorithm when distinguishing between young and old platelets. One point of contention concerning the field of platelet aging is whether platelet size changes with age. Although there is some evidence linking mean platelet volume to thrombotic risk [46,47], there are also contradictory data suggesting that the 2 variables are independent of one another [12,48,49]. The hypothesis that young platelets are larger was originally proposed in the 1960s [50,51]; however, by the mid-1980s, several studies were published reporting no correlation between platelet age and size [49]. Regardless, a caveat of using SYTO-13 dye as a surrogate marker of platelet age is that larger platelets may have more mRNA and subsequently take up more dye and appear brighter, skewing sorting and subsequent analysis [52]. However, previous work from our group using a similar mRNA dye noted variations in megakaryocyte-derived mRNAs such that young and old platelets would have to vary 32- to 64-fold in size for such a relationship to hold [33]. Similarly, it is also notable that SSC-A, generally taken as an indicator of internal complexity (ie, granularity) of a cell, is a highly important discriminator of young vs old platelets. This finding is directly in line with our observations that platelets lose approximately 50% of their total protein content and mitochondria as they age in the circulation [33]. Similarly, in turn this parameter may also reflect the potential density of a platelet, a measure that studies have suggested is an accurate indicator of platelet age [[53], [54], [55]].

For unseen samples, ML-based identification of young and old platelets was 76% accurate in vehicle control samples. This accuracy was maintained in platelets treated with PAR-4 or U46619 but decreased to 67% in platelets treated with ADP. Notably, identification of ADP-stimulated platelets was primarily determined by 4 parameters, while for all other conditions, 6 to 7 parameters were used, indicating that perhaps the composition of this panel could be altered for improved accuracy. Conversely, predictive accuracy was higher in samples treated with TRAP-6 or CRP-XL, at 83% and 84%, respectively. In addition, CD62P was more highly weighted as an additional discriminatory parameter in these samples, which reflects the well-described greater thrombotic potential of young platelets and their higher CD62P degranulation [12,56].

Finally, we applied our ML-based identification of young and old platelets back to our healthy platelet populations. This analysis demonstrated that young platelets, as defined by ML in each condition, were consistently associated with platelet clusters carrying higher levels of activation markers, in accordance with our earlier reports [12,33]. This suggests that ML algorithms employing FSC-A and SSC-A, in addition to surface markers, can be used to further discriminate platelet subpopulations in healthy individuals based on circulatory age.

Existing flow cytometry technology is starting to be replaced by machines incorporating full-spectrum measures and algorithm-based unmixing of contributing fluorophores. This will make larger multiparameter measurements of samples increasingly accessible to researchers. However, a key question will be the relevance and feasibility for clinical use as multiparametric measurements of platelets or aggregates potentially offer the capability of more accurate diagnoses or monitoring of platelet disorders and thrombotic risk. As we demonstrate here, this phenotypic approach is suitable for staining and analyzing very low volumes of WB, which we believe are important advantages to overcome the logistical and economical hurdles to more routine clinical use. An alternative approach to achieve this goal is the development of label-free analysis of samples, often based on image analysis [57]. Both approaches offer the capability to identify and differentiate subtle features. Indeed, with companies already developing spectral based imaging flow cytometers, it will most likely become possible to apply the 2 approaches simultaneously to analyze samples. At the heart of each approach is the application of ML, which is most powerful when informed by large numbers of samples to account for natural physiological variation. Achieving this will require acquisition of samples across multiple sites, which will also necessitate the development of standardized protocols.

In conclusion, we present a 16-parameter, flow cytometry–based assay coupled to powerful bioinformatic approaches to undertake unparalleled, deep phenotyping of platelets and their functionality. The incorporation of ML into this workflow provides impartial analysis and predictive capability at a by-platelet and by-individual level. We posit that this approach combining surface and physical markers will be highly valuable in phenotyping platelet subpopulations and studying platelet populations in disease or pathologic states.
