# Supplementary material for: Multiparameter phenotyping of platelets and characterization of the effects of agonists using machine learning
Source: Res Pract Thromb Haemost. 2024 Jul 22;8(5):102523. doi: 10.1016/j.rpth.2024.102523 (PMC11381873; doi:10.1016/j.rpth.2024.102523)

Multi-parameter phenotyping of platelets and characterisation of the effects of agonists using machine learning

Ami Vadgama[1], James Boot PhD[2], Nicola Dark[1], Harriet E. Allan PhD[1], Charles A. Mein PhD[2], Paul C. Armstrong PhD*[1], Timothy D. Warner PhD*[1]

[1]Centre for Immunobiology, [2]Genome Centre; Blizard Institute, Faculty of Medicine and Dentistry, Queen Mary University of London, London, United Kingdom

*PCA and TDW contributed equally to this work

Short title: Platelet immunophenotyping using machine learning

Corresponding author: Dr Paul C. Armstrong, p.c.armstrong@qmul.ac.uk, Centre for Immunobiology, Blizard Institute, Faculty of Medicine and Dentistry, Queen Mary University London, 4 Newark Street, London, E1 2AT, United Kingdom

Supplementary Data

Supplementary Figure S1: Similarity Indices for panel fluorophores with complexity index score as calculated using proprietary Cytek online support tool.

Supplementary Figure S2: Gating strategy for positive identification of platelets.

Supplementary Figure S3: Concentration-response curves of platelet activation in response to (A) TRAP-6 (0.1-10 µM), (B) PAR-4 (3-100 µM), (C) CRP-XL (0.03-3 µg/ml), (D) ADP (0.3-30 µM), and (E) U46619 (0.1-10 µM) measured by PAC-1 binding and CD62P exposure. Agonist concentrations chosen for subsequent assays highlighted within red boxes. Data are shown as MFI ±SEM or % positive ±SEM (n=3-4) and analysed using non-linear regression.

Supplementary Figure S4: Interindividual variation of individual markers in each tested condition. Coefficient of Variation (CV, %) calculated across all donors (n=21) comparing median fluorescence value of each 10,000 platelets per condition per donor.

Supplementary Figure S5: Composite tSNE of all donor platelets (n=20-21) for (A) vehicle, (B) ADP, (C) TRAP-6, (D) U46619, (E) PAR-4, and (F) CRP-XL.

Supplementary Figure S6: High dimensionality analysis of platelet sub-populations using a clustering model in (A) resting/vehicle-treated, (B) ADP-treated, (C) TRAP-6-treated, (D) U46619-treated, (E) PAR-4-treated, and (F) CRP-XL-treated platelets. Data presented as clustered tSNE population division (left panel), corresponding donors making up each cluster (middle panel), and heatmap breakdown of fold-change in marker expression in each cluster (low=white, high=purple; right; right panel; n=20-21).

Supplementary Figure S7: Changes in surface marker expression in “young” and “old” resting and activated platelets expressed as raw MFI (left) and change in MFI (right). Data are shown as MFI ±SEM (n=8) and analysed using 2-way ANOVA or mixed-effects analysis with Tukey’s multiple comparisons test.

Supplementary Figure S8: Changes in individual surface marker expression of platelets in basal and activated whole blood expressed as (A) raw MFI ±SEM, (B) a heatmap based on MFI values, and (C) a heatmap based on MFI log fold-changes. Data were analysed using a one-way ANOVA with a Dunnett test to correct for multiple comparisons (n=6).

Supplementary Figure S9: Comparative performance of assay and panel performed in diluted PRP or whole blood. Individual surface marker expression of platelets expressed as raw MFI ±SEM were analysed using 2-way ANOVA with Sidak’s multiple comparisons test (n=6).

Supplementary Table S1: Details of panel antibodies.

Marker	Alias	Role	Fluorophore	Clone	Dilution factor		Laser (Channel)	Supplier (Catalogue number)
					PRP	WB		
CD107a	LAMP-1	Lysosome degranulation	PE	H4A3	1:200	1:100	Yellow/Green (YG1)	BioLegend (328608)
CD29	GPIIa	Adhesion integrin	BUV737	MAR4	1:200	1:200	Ultraviolet (UV14)	BD Biosciences (748618)
CD31	PECAM-1	α-granule protein; platelet-endothelial cell interaction	BV785	WM59	1:200	1:100	Violet (V15)	BioLegend (303148)
CD36	GPIV	α-granule protein; Scavenger receptor	BV605	CB38/NL 07	1:70	1:40	Violet (V10)	BD Biosciences (563518)
CD41	GPIIb,	Fibrinogen binding integrin	APC-Cy7	HIP8	1:100	1:100	Red (R7)	BioLegend (303716)
CD61	GPIIIa,		BUV395	RUU-PL7F12	1:70	1:70	Ultraviolet (UV2)	BD Biosciences (745717)
CD42a	GPIX	Adhesion integrin; binds vWF	eFluor 450	GR-P	1:30	1:30	Violet (V3)	Thermo Fisher Scientific (48-0428-42)
CD42b	GPIbα		BV650	HIP1	1:30	1:30	Violet (V11)	BioLegend (303926)
CD62P	P-selectin	α-granule protein; platelet-endothelial/ leukocyte binding	BB700	AK-4	1:100	1:70	Blue (B9)	BD Biosciences (566565)
CD63	LAMP-3	δ-granules, lysosomes marker	PE-Cy7	H5C6	1:70	1:70	Yellow/Green (YG9)	Thermo Fisher Scientific (25-0639-42)
CD9	TSPAN29	Tetraspanin; modulate collagen response/ GPIIb/IIIa	BV510	M-L13	1:70	1:40	Violet (V7)	BD Biosciences (563640)
CLEC-2	CLEC-1b	Thromboinflammatory receptor	BV711	219133	1:70	1:40	Violet (V13)	BD Biosciences (748142)
GPVI		Collagen receptor	AF647	HY101	1:200	1:200	Red (R2)	BD Biosciences (564701)
PAC-1		Binds activated confirmation of receptor GPIIb/IIIa integrin	FITC	PAC-1	1:10	1:7	Blue (B2)	BD Biosciences (340507)

Supplementary Figure S1

Similarity™ Indices

Configuration:5L 16UV-16V-14B-10YG-8R

	BV510	BV785	BV605	eFluor 450	BV650	PE-Cy7	PE	Alexa Fluor 647	BV711	BB700	BUV737	APC-Cy7	BUV395	FITC
BV510	1													
BV785	0.03	1												
BV605	0.4	0.07	1											
eFluor 450	0.27	0.07	0.06	1										
BV650	0.16	0.15	0.53	0.08	1									
PE-Cy7	0	0.17	0.03	0	0.04	1								
PE	0.12	0	0.28	0.01	0.05	0.02	1							
Alexa Fluor 647	0	0.01	0.03	0	0.18	0.03	0.01	1						
BV711	0.06	0.49	0.17	0.08	0.45	0.11	0.01	0.18	1					
BB700	0.05	0.21	0.16	0	0.37	0.17	0.06	0.33	0.53	1				
BUV737	0.02	0.25	0.05	0	0.13	0.14	0	0.22	0.41	0.3	1			
APC-Cy7	0	0.23	0.02	0	0.06	0.29	0	0.18	0.19	0.16	0.32	1		
BUV395	0.02	0	0.01	0	0	0	0.01	0	0	0	0.03	0	1	
FITC	0.06	0	0.01	0.01	0	0	0.09	0	0	0.02	0	0	0.01	1

Complexity™ Index: 3.39

Supplementary Figure S2

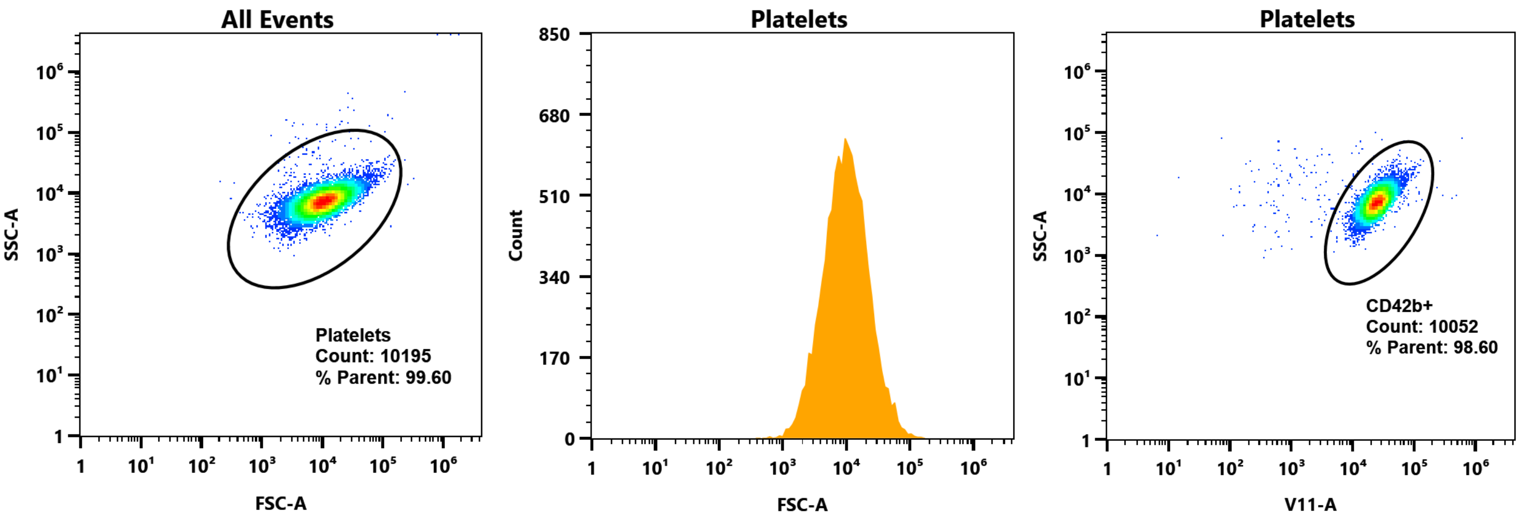

Supplementary Figure S3

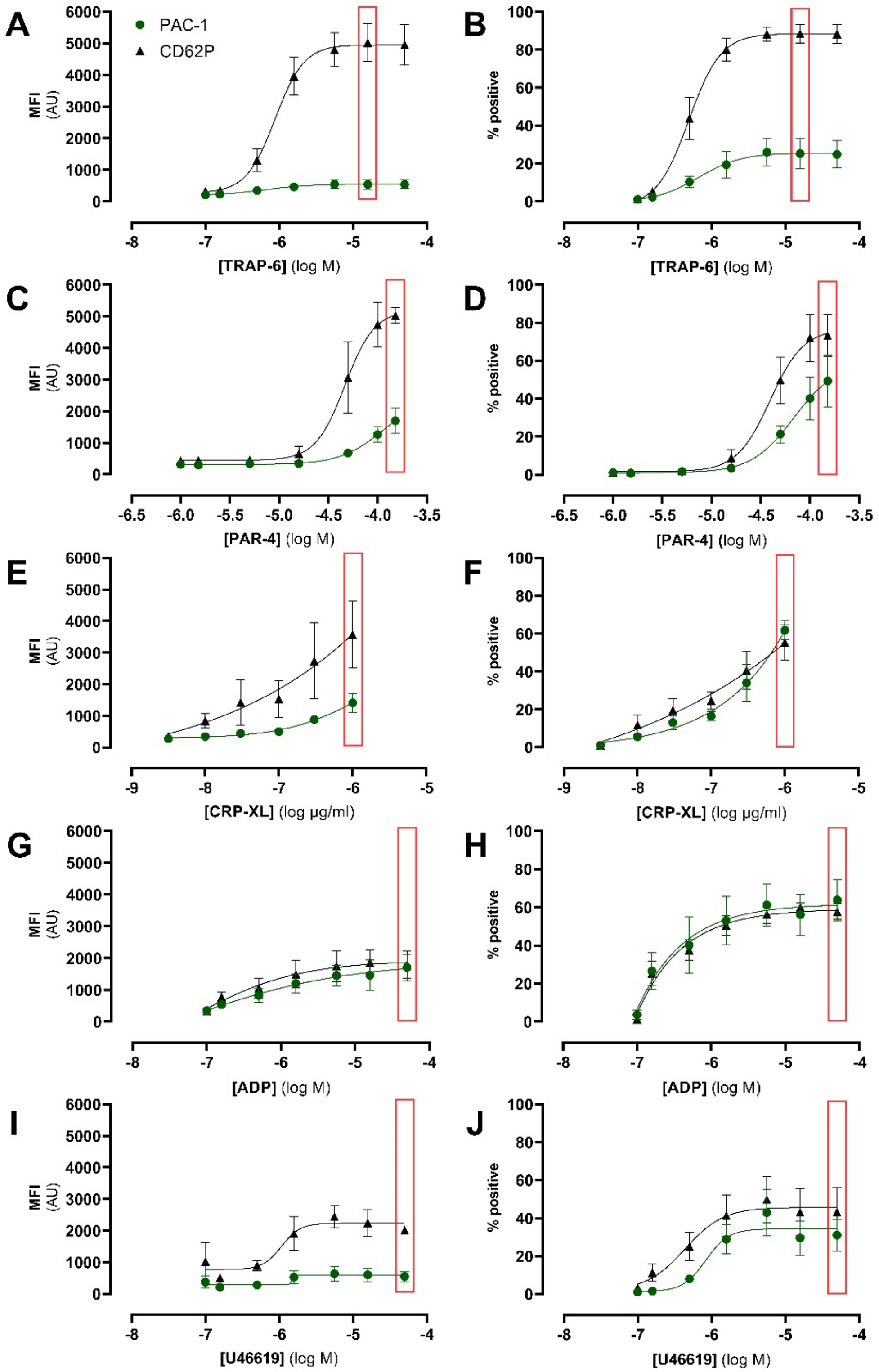

Supplementary Figure S4

Interindividual variability; CV (%)

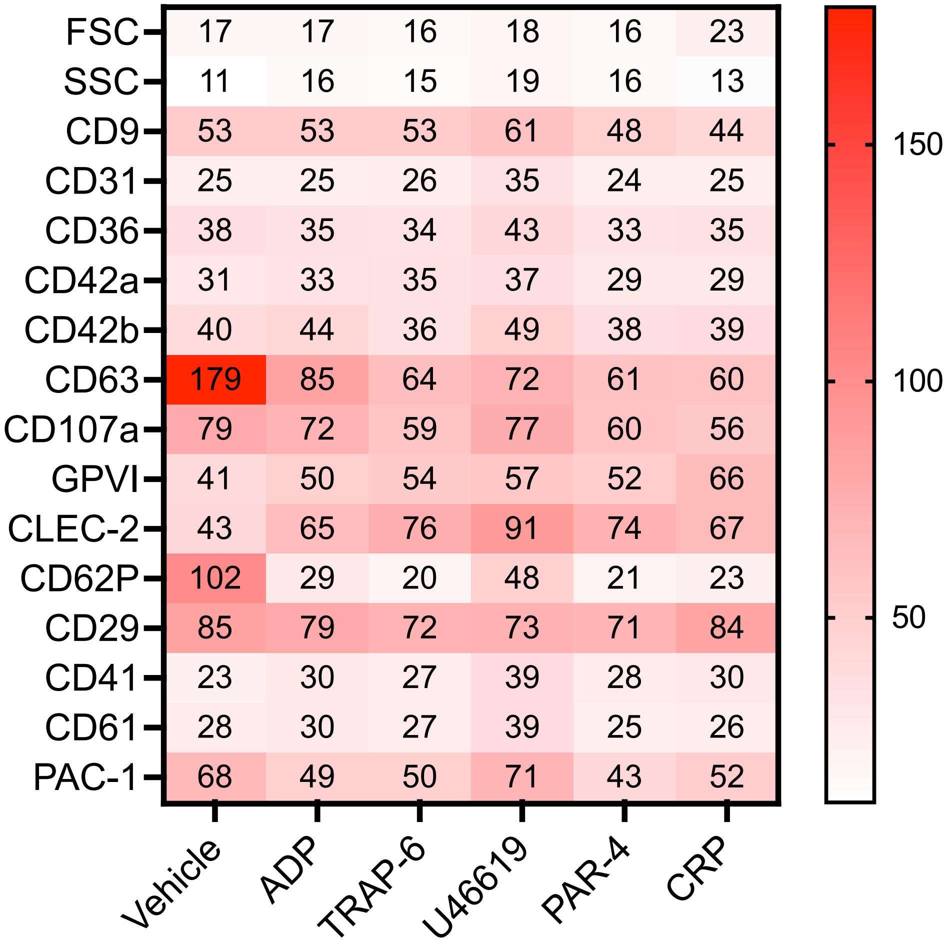

Supplementary Figure S5

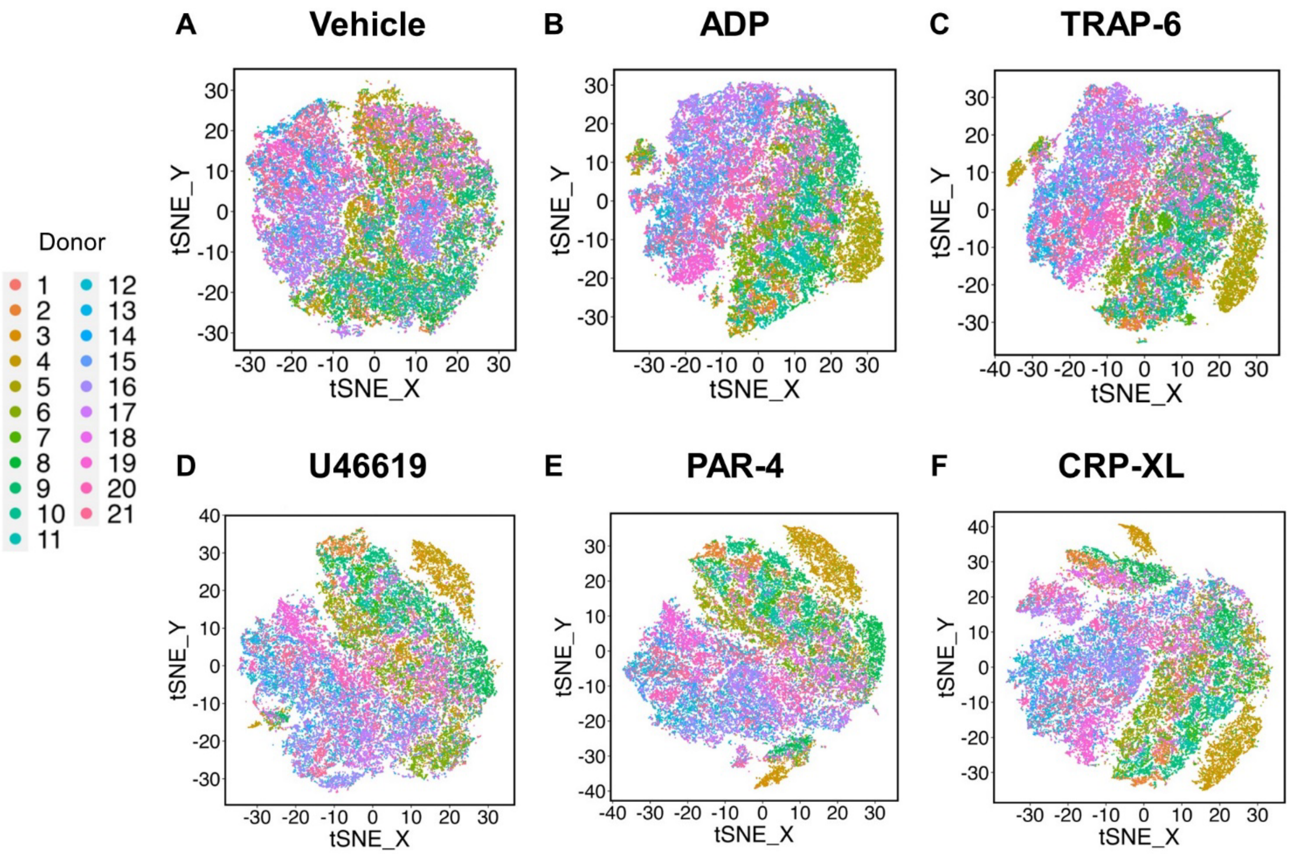

Supplementary Figure S6

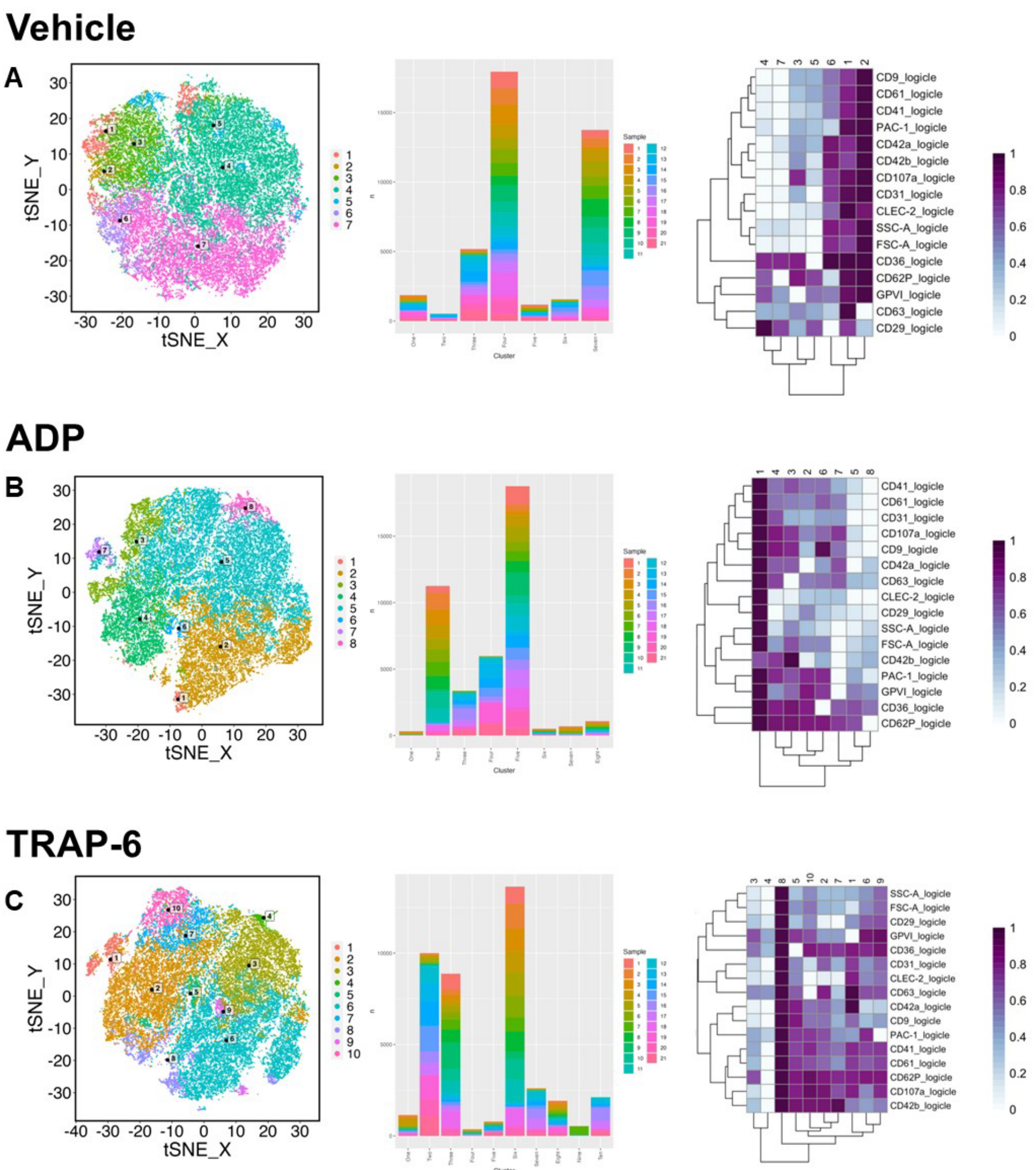

Supplementary Figure S6 cont.

U46619

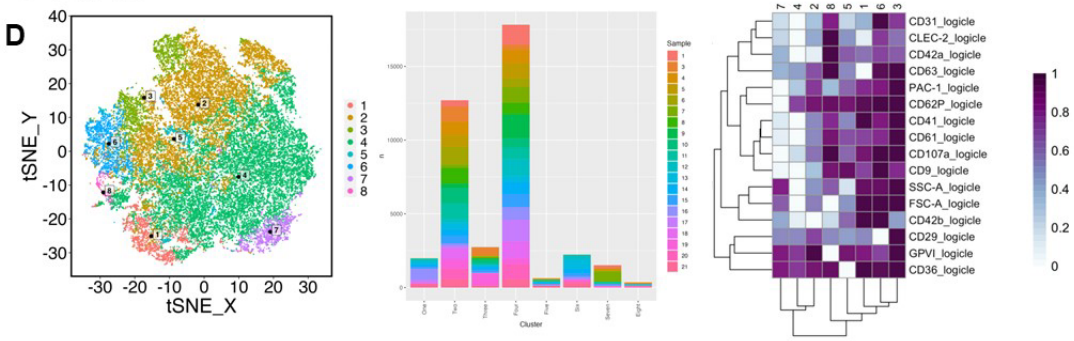

PAR-4

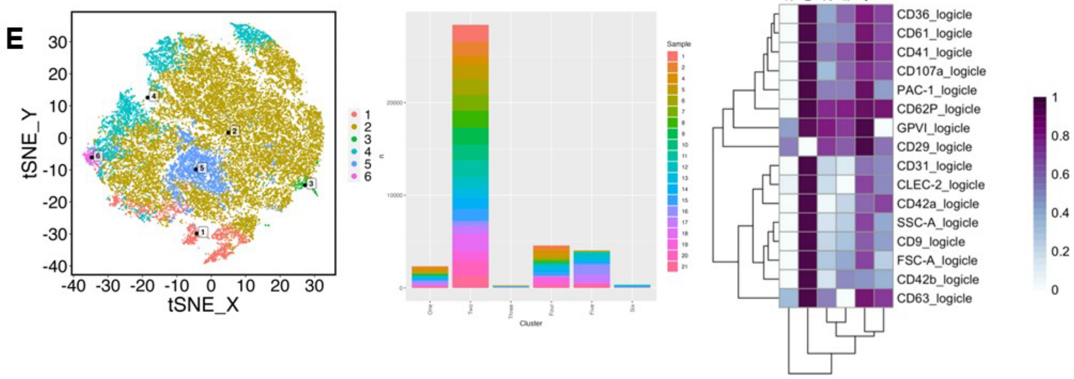

CRP-XL

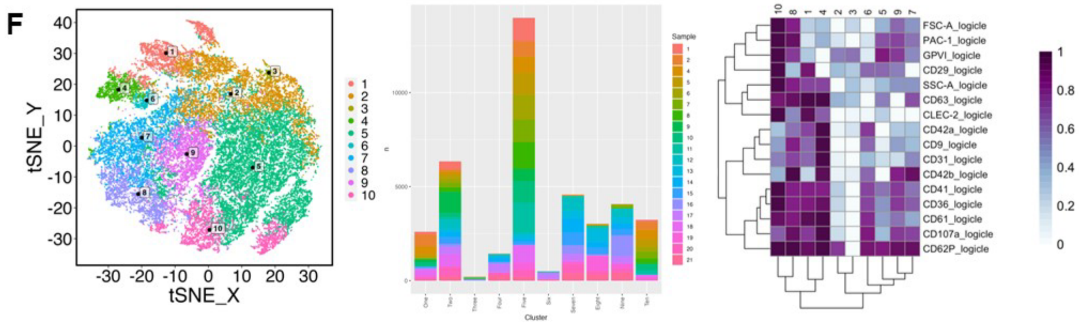

Supplementary Figure S7

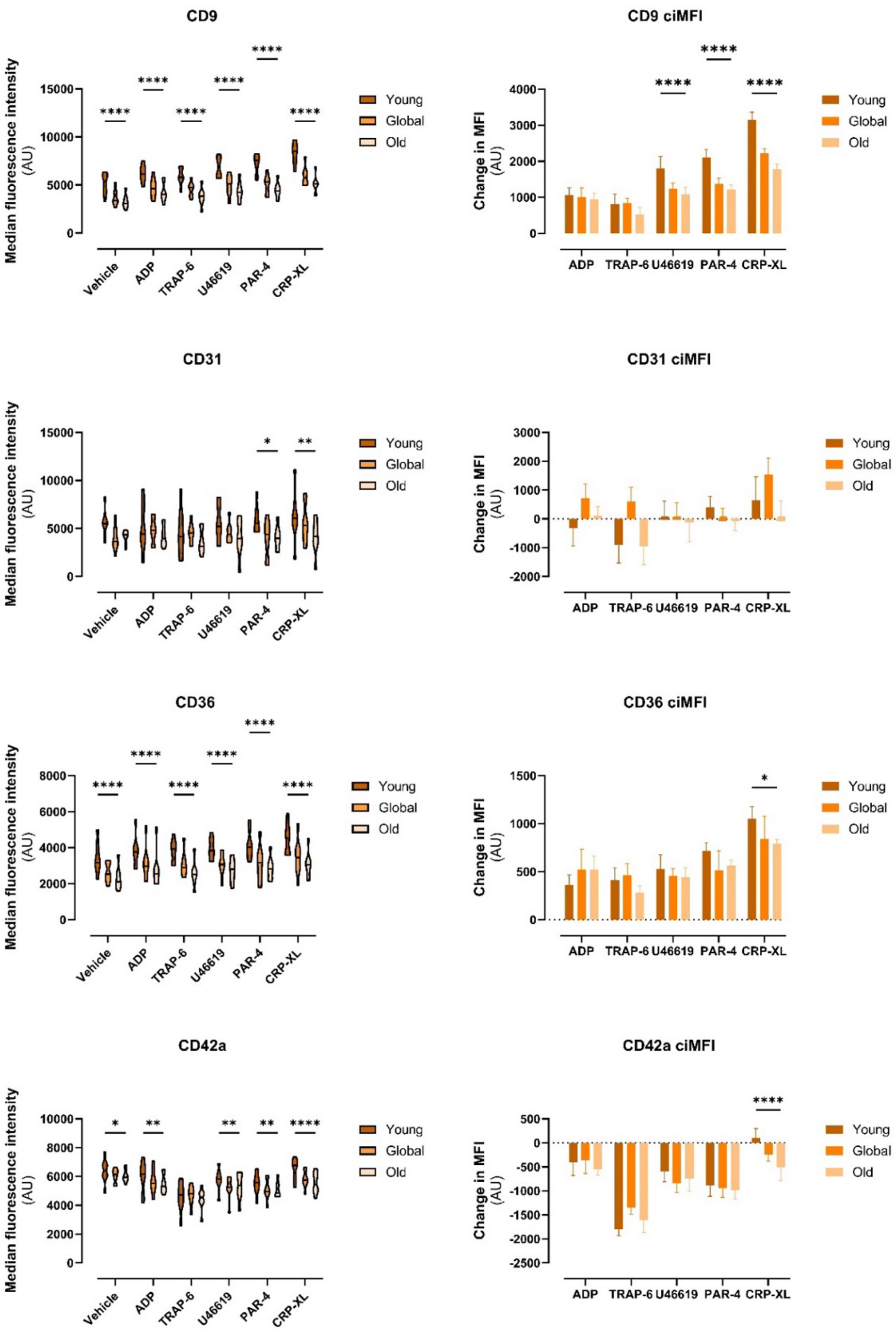

Supplementary Figure S7 cont.

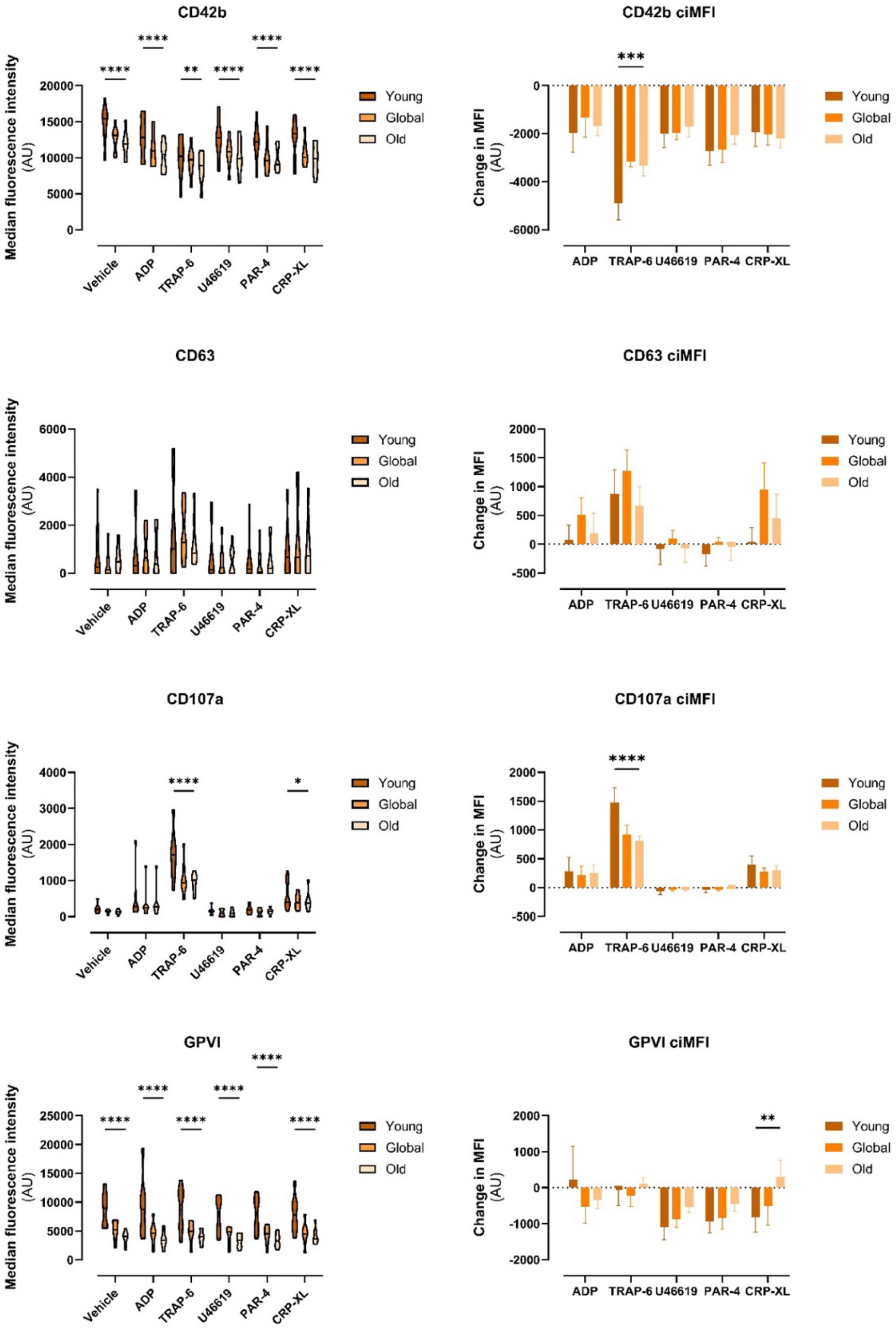

Supplementary Figure S7 cont.

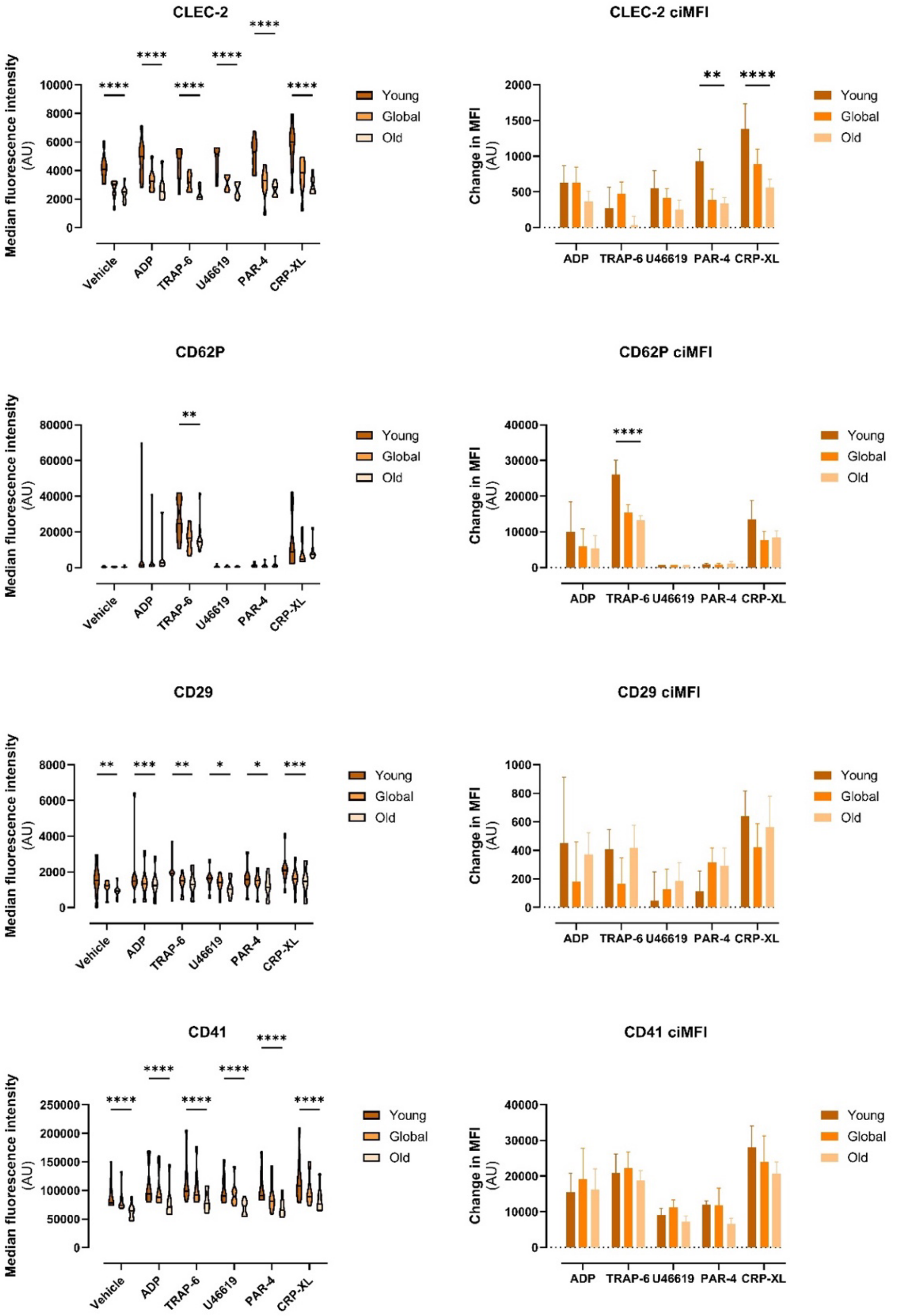

Supplementary Figure S7 cont.

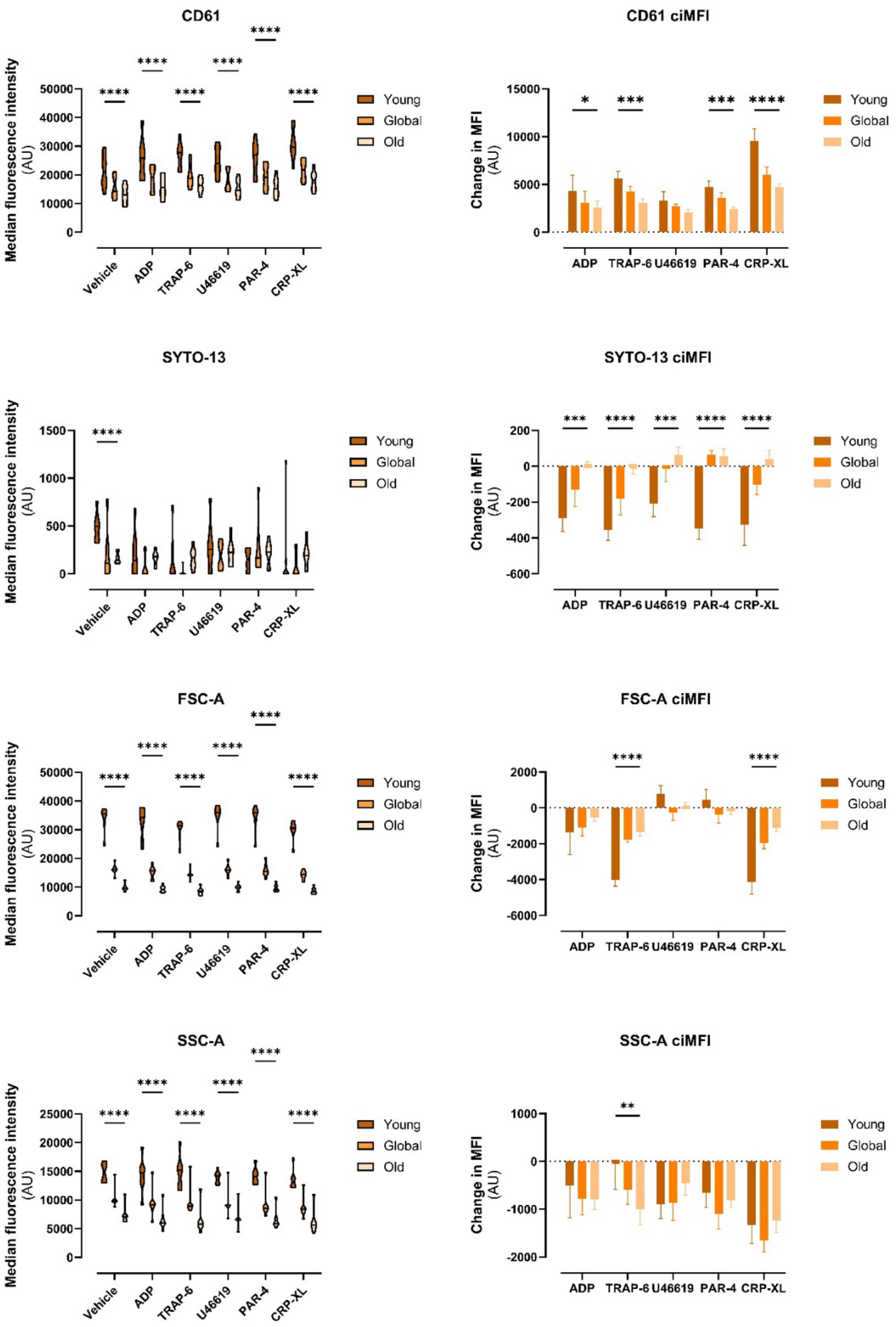

Supplementary Figure S8

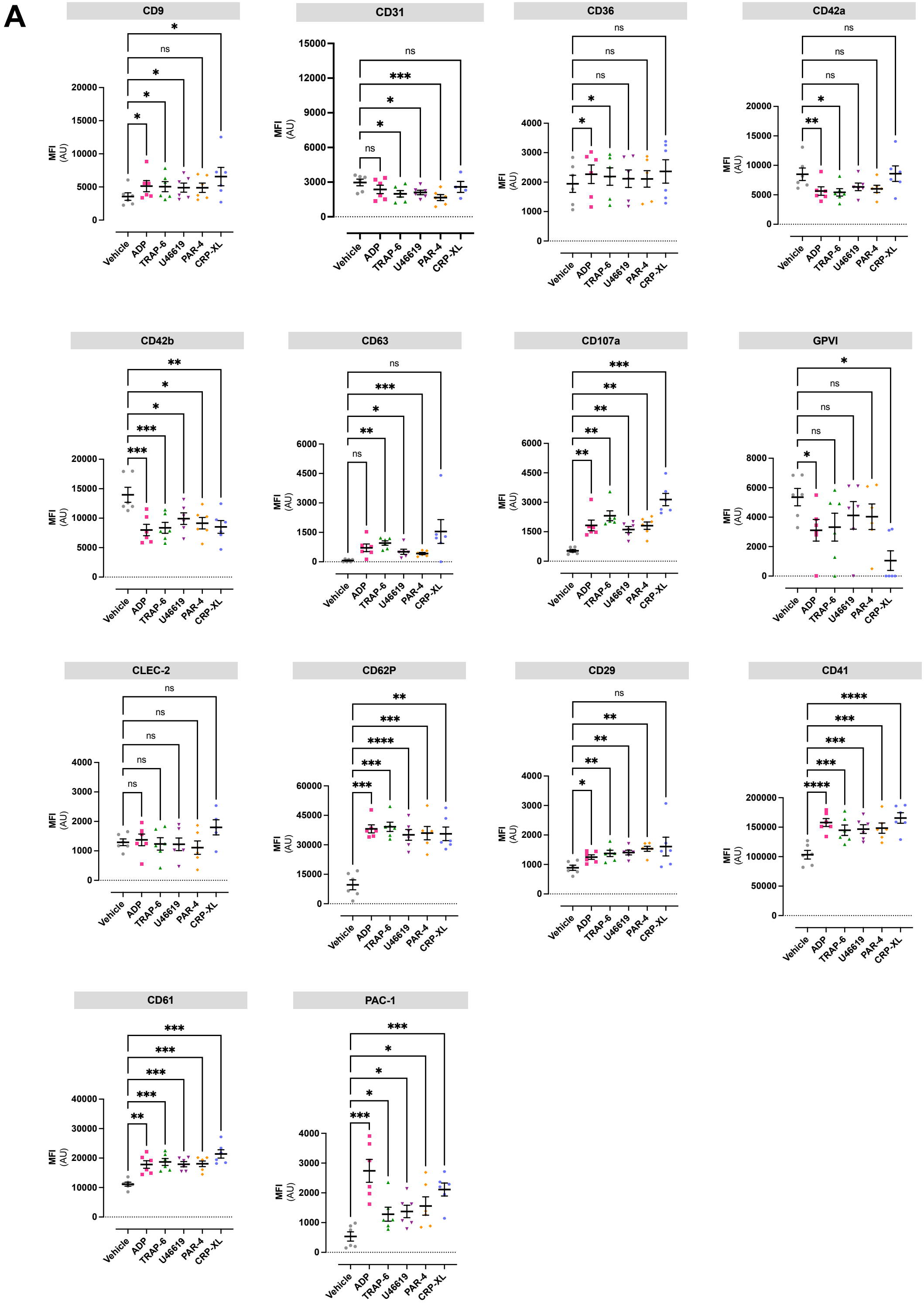

Supplementary Figure S8 cont**.**

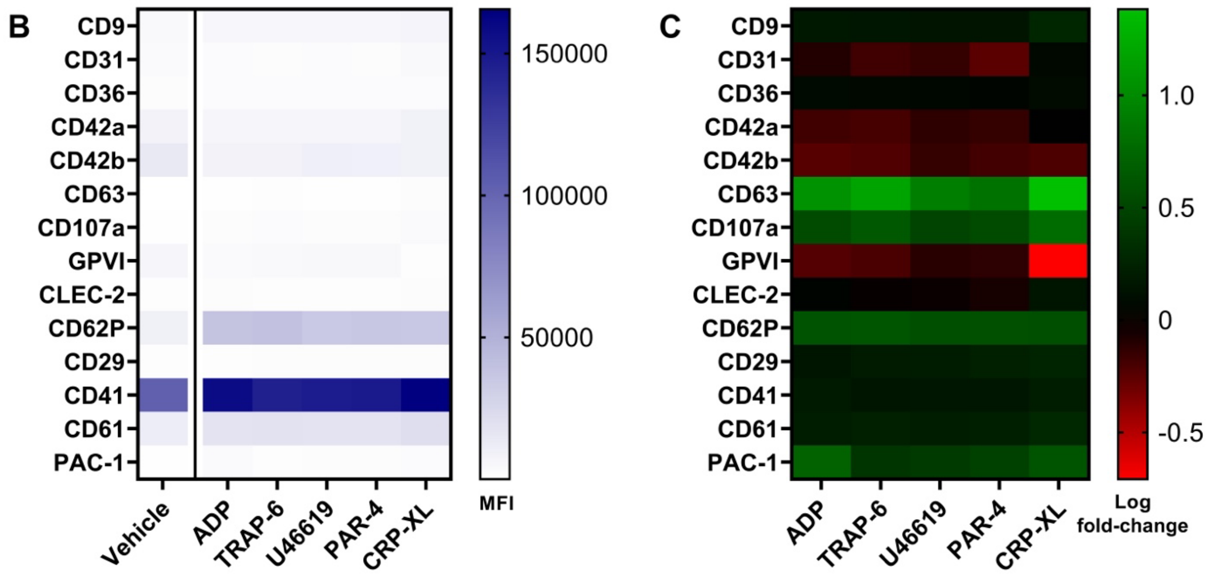

Supplementary Figure S9

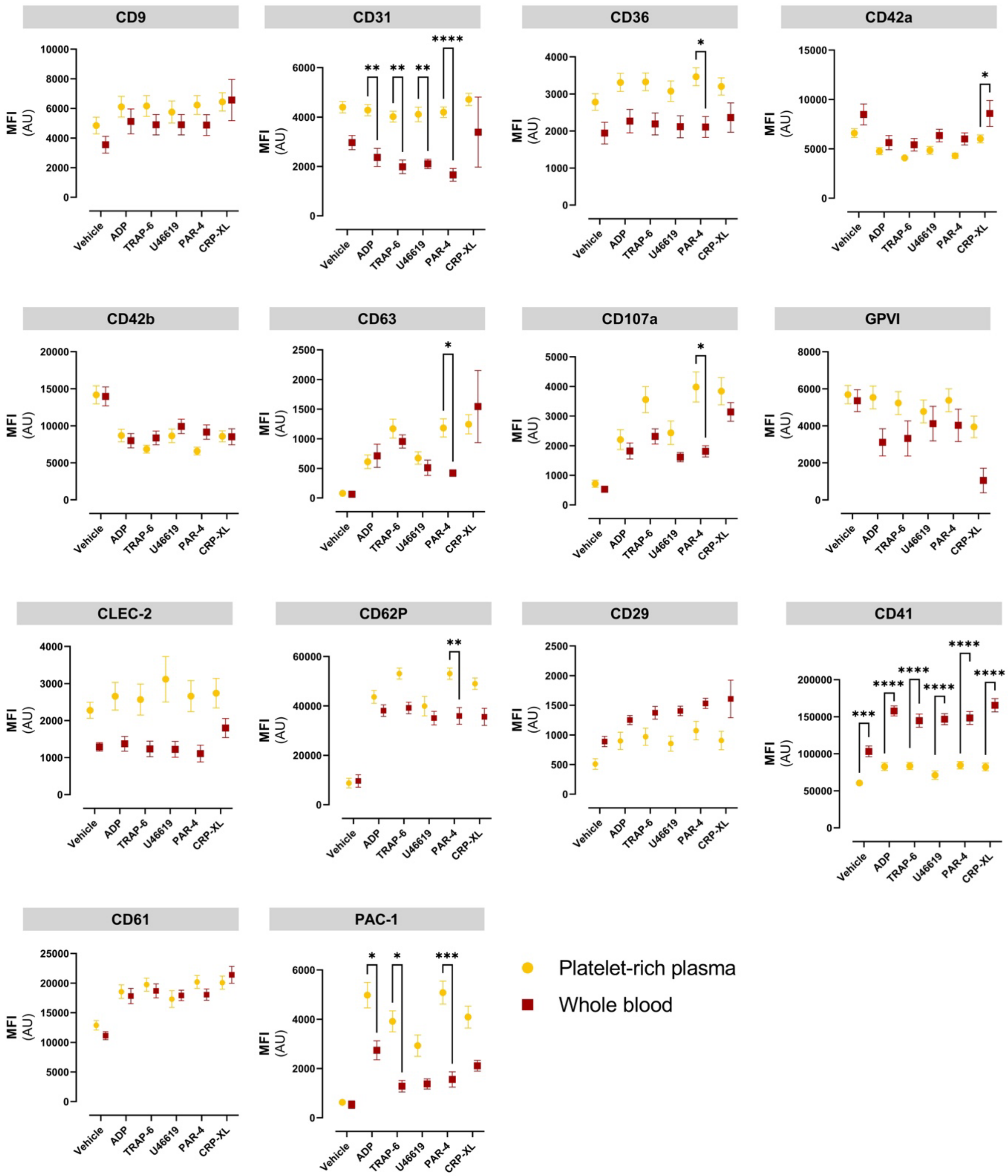

Supplement: Supplementary Material [file mmc1.pdf]
